# The effects of ProAlgaZyme novel algae infusion on metabolic syndrome and markers of cardiovascular health

**DOI:** 10.1186/1476-511X-6-20

**Published:** 2007-09-05

**Authors:** Julius Oben, Ebangha Enonchong, Dieudonne Kuate, Dora Mbanya, Tiffany C Thomas, DeWall J Hildreth, Thomas D Ingolia, Michael S Tempesta

**Affiliations:** 1 Laboratory of Nutrition and Nutritional Biochemistry, Department of Biochemistry, BP 812, University of Yaoundé I, Yaoundé, Cameroon; 2 Health Enhancement Products, Inc. 7740 East Evans Road, Scottsdale, AZ USA; 3 Faculty of Medicine and Biomedical Sciences, University of Yaoundé I, Cameroon

## Abstract

**Background:**

Metabolic Syndrome, or Syndrome X, is characterized by a set of metabolic and lipid imbalances that greatly increases the risk of developing diabetes and cardiovascular disease. The syndrome is highly prevalent in the United States and worldwide, and treatments are in high demand. ProAlgaZyme, a novel and proprietary freshwater algae infusion in purified water, has been the subject of several animal studies and has demonstrated low toxicity even with chronic administration at elevated doses. The infusion has been used historically for the treatment of several inflammatory and immune disorders in humans and is considered well-tolerated. Here, the infusion is evaluated for its effects on the cardiovascular risk factors present in metabolic syndrome in a randomized double-blind placebo-controlled study involving 60 overweight and obese persons, ages 25–60. All participants received four daily oral doses (1 fl oz) of ProAlgaZyme (N = 22) or water placebo (N = 30) for a total of 10 weeks, and were encouraged to maintain their normal levels of physical activity. Blood sampling and anthropometric measurements were taken at the beginning of the study period and after 4, 8 and 10 weeks of treatment. Eight participants did not complete the study.

**Results:**

ProAlgaZyme brought about statistically significant (p < 0.001) reductions in the following: weight, body fat, total cholesterol, LDL-cholesterol, triglycerides, C-reactive protein and fasting blood glucose levels, accompanied by a significant (p < 0.001) increase in HDL-cholesterol levels over the 10-week study period. The infusion was well-tolerated and no side effects were noted.

**Conclusion:**

ProAlgaZyme (4 fl oz daily) consumption resulted in significant reductions in weight and blood glucose levels, while significantly improving serum lipid profiles and reducing markers of inflammation, thus improving cardiovascular risk factors in overweight and obese subjects over a course of 10 weeks with an absence of adverse side effects.

**Trial Registration:**

US ClinicalTrials.gov NCT00489333

## Background

Multiple risk factor syndrome or metabolic syndrome (i.e. the coexistence of several risk factors for artherosclerosis, including hyperglycemia, dyslipidemia and hypertension in the same individual) is a growing medical problem in industrialized countries [1,2], with obesity being the central and causal component.

Obesity, which is defined as an excess deposition of fat in body fat stores, is determined using the Body Mass Index or BMI scale. It could also be determined by bioelectrical impedance methods, which gives an estimate of the amount of fat stores in the body. The distribution of these fat stores (android or gynoid) is known to be an independent risk factor in obesity-related diseases.

In the United States, over 60% of the adult population is now overweight or obese [3] and 47 million people have metabolic syndrome, which will soon overtake cigarette smoking as the number one risk factor for heart disease [4,5]. Obesity and its related complications were considered for a long time to be limited to industrialized developed countries. This is however no longer the case, with a high incidence now in developing countries who have undergone lifestyle changes which increase the incidence of these diseases.

Obesity has been shown to contribute to high serum cholesterol, low HDL cholesterol and hyperglycemia, all of which increase the chances of cardiovascular disease (CVD) [6-8]. Correlations between central obesity and high blood pressure, high blood cholesterol, low levels of high density lipoprotein cholesterol and high fasting blood glucose levels have been shown for both sexes in various racial and ethnic groups [6-10].

The complications of obesity are worsened by oxidative stress (imbalance between pro-oxidants and antioxidants in favor of pro-oxidants). Recent research has shown that long-term caloric restriction promotes longevity and chronic overeating and obesity results in a condition that mimics the aging process, which explains the higher prevalence of inflammation and free radicals, and metabolic and cardiovascular diseases in overweight individuals. Obesity may induce systemic oxidative stress, and increased oxidative stress in accumulated fat is one of the underlying causes of dysregulation of adipocytokines and development of metabolic syndrome [6].

The increased chance of CVD and type 2 diabetes requires therapeutic consideration for the vast numbers of overweight/obese persons now at high risk for these diseases [11]. The current International Diabetes Foundation recommendations for preventing or delaying the development of diabetes include both primary and secondary interventions. The former emphasizes lifestyle changes such as calorie restriction and increased physical activity, and the latter (for people at high risk for CVD) uses pharmacological agents [12] that specifically target individual components of metabolic syndrome [12-17]. When used by obese patients in combination with dietary regimes or alone, these agents can produce some weight loss and some prevention or reversal of accompanying complications. The role of pharmacotherapy, however, has been compromised by safety issues leading to the withdrawal of some medications from the market [18,19] or from advanced stages of clinical testing. Furthermore, agents that can address the underlying issues behind many of the complications associated with metabolic syndrome have yet to be developed.

This study investigated the effects of ProAlgaZyme algae infusion ("PAZ") vs. placebo on indicators of cardiovascular health by assessing markers of inflammation and oxidative stress, blood sugar, weight and blood lipids in overweight and obese people. ProAlgaZyme (PAZ) is a freshwater algae infusion that has been consumed as a liquid dietary supplement for over 3 decades. PAZ was first marketed as Lebenszeit^TM^, meaning "Lifetime" in German, in the 1980's by Ponce de Leon Medical Development Corp. in the U.S., in reference to the legendary fountain of youth. The product became known as ProAlgaZyme or "PAZ" in 2003. The infusion is an aqueous fermentation product of a proprietary blend of freshwater organisms including green algae discovered over 30 years ago within a natural source, and has since been cultivated under controlled conditions. ProAlgaZyme oral liquid dietary supplement, the filtrate of the fermentation process, typically has less than 100 ppm total dissolved solids consisting of approximately 90% salts (free of heavy metals at a detection limit of <0.1 ppm) and a unique blend of organic constituents currently undergoing full characterization.

Anecdotal reports in the U.S. of improvements in symptoms of inflammatory and metabolic conditions after PAZ consumption led to our interest in evaluating PAZ's potential to positively affect factors associated with metabolic syndrome and cardiovascular health. Particularly relevant to this study were anecdotal reports of improvements in high sensitivity C-reactive protein, blood sugar metabolism and body weight, and increases in energy. Patients with metabolic syndrome were specially suited as subjects in this study, as this diagnosis includes both metabolic and inflammatory components. The human clinical study results are reported here.

### Objectives of study

The main objective of the study was to establish the role of ProAlgaZyme algae infusion (PAZ) vs. placebo to control inflammation and promote cardiovascular benefits in overweight and obese people. Specific objectives were to determine the effects of ProAlgaZyme vs. placebo on body weight, BMI, blood lipids, fasting blood glucose levels and markers of inflammation over a 10-week period.

## Results

ProAlgaZyme had a significant effect on the various parameters studied in metabolic syndrome patients over a 10 week period (Table 1). The raw data at 4, 8 and 10 weeks are in additional file 1: MetSyn_Data.xls.

**Table 1:** The effect of ProAlgaZyme on various parameters related to metabolic syndrome. Bolded values represent mean Δ after 10* weeks of treatment ± (1.96*SEM) for a 95% confidence level. Values in parentheses are mean starting values (at T = 0) ± (1.96*SEM). Significance indicates comparison of changes after 10* weeks of treatment with PAZ to placebo. A negative value indicates a decrease, while a positive value indicates an increase over the test period. *Values for RBC sedimentation, triglycerides, LDL-C and blood pressure are after 8 weeks of treatment. NS = Not significant.

**Parameters**	Δ **ProAlgaZyme **(T = 0)	Δ **Placebo **(T = 0)	**Significance**
Weight (kg)	**-4.18 ± 0.76** (86.70 ± 2.31)	**-1.20 ± 0.29** (86.34 ± 2.61)	P < 0.001
BMI (kg/m^2^)	**-1.51 ± 0.28** (31.13 ± 0.62)	**-0.20 ± 0.11** (31.95 ± 0.63)	P < 0.001
Body fat (%)	**-2.46 ± 3.51** (40.14 ± 0.79)	**-0.30 ± 0.17** (41.88 ± 1.11)	P < 0.001
Blood pressure (Sys^8^, mm Hg)	**-10.32 ± 2.69** (152.23 ± 4.41)	**-1.36 ± 2.18** (158.21 ± 4.39)	P < 0.01
Blood pressure (Dias^8^, mm Hg)	**-7.27 ± 2.50** (68.73 ± 2.58)	**-2.32 ± 1.84** (72.82 ± 2.69)	P < 0.001
Insulin (uIU/mL)	**1.28 ± 2.34** (23.48 ± 2.23)	**-1.01 ± 1.57** (23.02 ± 3.18)	NS
Fasting blood glucose (mg/dL)	**-18.50 ± 5.56** (98.72 ± 5.65)	**-0.58 ± 2.11** (98.41 ± 3.41)	P < 0.001
Total cholesterol (mg/dL)	**-64.84 ± 9.68** (201.94 ± 3.92)	**-5.45 ± 2.51** (204.17 ± 3.87)	P < 0.001
HDL cholesterol (mg/dL)	**15.37 ± 3.04** (37.02 ± 1.07)	**-2.98 ± 1.53** (38.76 ± 1.44)	P < 0.001
LDL cholesterol^8 ^(mg/dL)	**-40.63 ± 9.58** (131.32 ± 3.73)	**-0.69 ± 2.79** (131.45 ± 4.15)	P < 0.001
Triglycerides^8 ^(mg/dL)	**-18.77 ± 6.18** (167.95 ± 7.35)	**-1.96 ± 4.52** (166.67 ± 4.64)	P < 0.001
RBC sed. rate^8 ^(mm/h)	**-3.05 ± 0.78** (14.23 ± 0.58)	**-0.25 ± 0.65** (14.36 ± 0.68)	P < 0.001
C-reactive protein (mg/L)	**-6.20 ± 0.92** (10.83 ± 0.51)	**-0.83 ± 0.49** (11.41 ± 0.67)	P < 0.001
Interleukin-6 (pg/mL)	**-3.62 ± 1.03** (14.40 ± 2.01)	**-0.03 ± 0.26** (12.65 ± 1.20)	P < 0.001
TNF-a (pg/mL)	**-0.65 ± 0.66** (11.22 ± 1.14)	**-0.08 ± 0.44** (10.95 ± 0.84)	P < 0.05

A total of 52 overweight or obese participants aged 25 to 60 years completed the study (87%). Eight patients dropped out of the study; three gave as a reason difficulty in maintaining the dosing schedule, 3 others did not give any reason, while 2 of them moved out of town. While no side effects were reported in either the dropouts or in those completing the trial, it is possible that unreported reactions to the ProAlgaZyme treatment led to the decision in this subgroup to stop participation. Mitigating against this possibility are results from a separate trial (submitted for publication), in which 20 HIV+ individuals received 20 ounces per day (5 times the dose used in this study) for a 12-week period, with no side effects observed or reported.

### Weight, BMI and body fat

**Figure 1:**
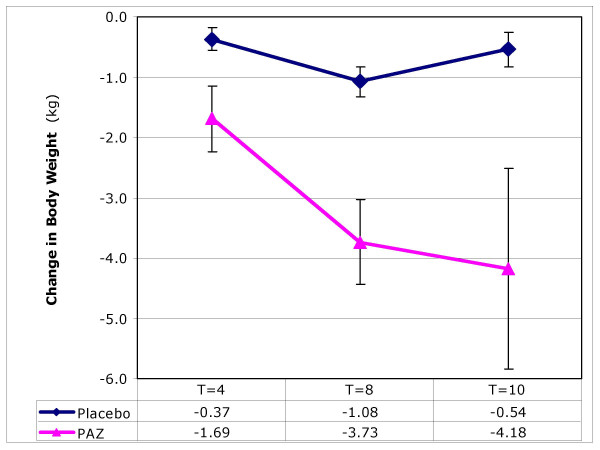
**The effect of ProAlgaZyme vs. placebo on body weight (kg)**. Values are mean change at 4, 8 and 10 weeks compared to baseline within 95% confidence limits (+/- 1.96*(standard error of the mean)).

After 10 weeks, participants receiving ProAlgaZyme had significantly (p < 0.001) greater reductions in body weight and consequently body mass index (BMI) compared to placebo (Figure 1). This reduction was paralleled by a significant decrease in the percentage of body fat over this period in the treatment group vs. placebo (Table 1).

### Blood pressure

After 8 weeks of treatment, PAZ significantly (p < 0.01 and p < 0.001) reduced the systolic and diastolic blood pressure of participants, respectively, compared to placebo (Table 1)

### Insulin and fasting blood glucose levels

Mean fasting blood glucose levels in metabolic syndrome patients were significantly (p < 0.001) lowered in the PAZ group compared to placebo (Table 1). Although modest increases in the circulating levels of insulin were observed in the treatment group, these were not significant.

### Blood lipids: total cholesterol, LDL-cholesterol, HDL-cholesterol and triglycerides

**Figure 2:**
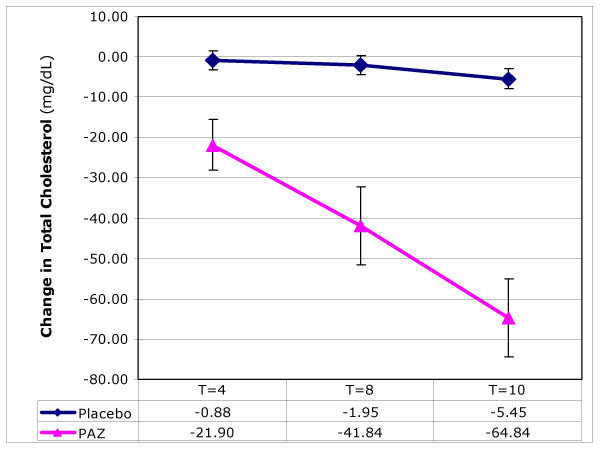
**The effect of ProAlgaZyme vs. placebo on total cholesterol (mg/dL)**. Values are mean change at 4, 8 and 10 weeks compared to baseline within 95% confidence limits (+/- 1.96*(standard error of the mean)).

**Figure 3:**
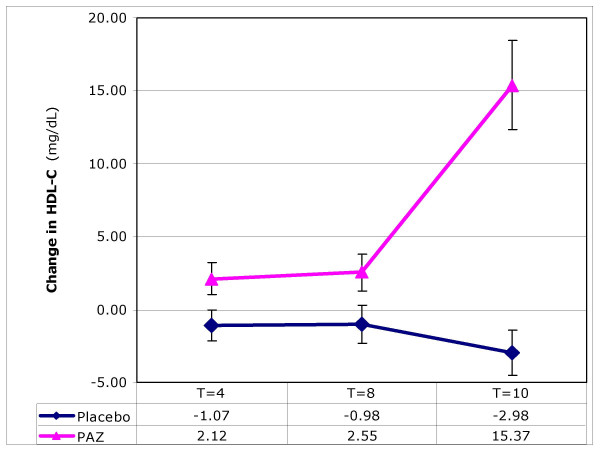
**The effect of ProAlgaZyme vs. placebo on HDL-cholesterol (mg/dL)**. Values are mean change at 4, 8 and 10 weeks compared to baseline within 95% confidence limits (+/- 1.96*(standard error of the mean)).

PAZ brought about significant changes in all lipid parameters measured. In the treatment group, significant (p < 0.001) reductions were observed in circulating total cholesterol (Figure 2), LDL-C and triglycerides over the 10-week test period (Table 1). These changes were accompanied by a significant (p < 0.001) increase in HDL-cholesterol (Figure 3) while a decrease was observed in the placebo group. Final values for LDL-C and triglycerides were determined at T = 8 weeks; others parameters were at T = 10 weeks.

### Red blood cell sedimentation, C-reactive protein, interleukin-6, TNF-a

**Figure 4:**
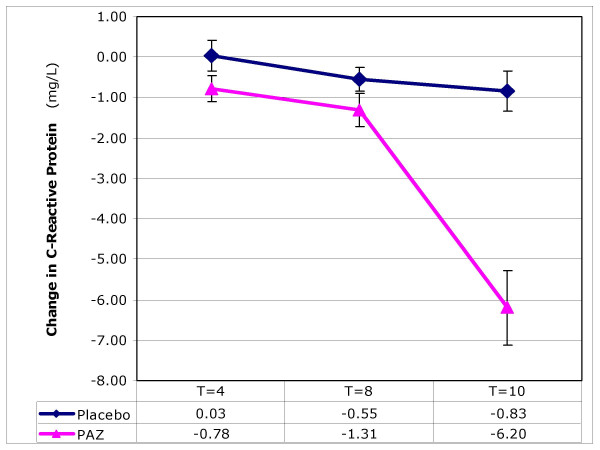
**The effect of ProAlgaZyme vs. placebo on C-reactive protein (mg/L)**. Values are mean change at 4, 8 and 10 weeks compared to baseline within 95% confidence limits (+/- 1.96*(standard error of the mean)).

There were significantly greater decreases (p < 0.001) compared to placebo in RBC sedimentation rate and concentrations of CRP (Figure 4) and IL-6 in test subjects after 10 weeks. Levels of TNF-alpha were less significantly (p < 0.05) altered.

### Time course of effects

Individual patient values, means and calculations of percentage change from baseline for each parameter are available in additional file 1: MetSyn_Data.xls. Exemplary data for changes as a function of time are shown graphically for body weight, total cholesterol, HDL-C, and CRP in Figures 1, 2, 3, 4, respectively. Figure 1 shows that the rate of change for body weight was greater than for the other parameters, especially CRP. This raises the possibility that the change in body weight is a primary effect and some or all of the other changes may be secondary effects.

## Discussion

In this study, we evaluated the effects of ProAlgaZyme algae infusion (PAZ) on indicators of metabolic syndrome and subsequent risk factors for cardiovascular disease. The results obtained showed that PAZ is efficient in improving multiple markers of metabolic syndrome including weight, blood lipids, fasting blood glucose and markers of inflammation, and may have a significant effect on cardiovascular health. The infusion is effective in reducing body weight in obese patients, with an average weight loss of 4.2 kg (9 lbs.) over 10 weeks. This reduction seems to be caused by the decrease in percentage of body fat observed throughout the study in the PAZ group, with an average loss of 2.6% body fat. Accumulation of body fat is tightly linked to non-insulin dependent diabetes mellitus (NIDDM). In this study, results also showed a hypoglycemic effect of PAZ, with an average reduction in fasting blood glucose of nearly 19% or 18.50 mg/dL. It would be interesting to investigate whether this effect could also be observed in people living with diabetes mellitus. Several hypotheses can be made in order to explain the mechanism of the weight/fat loss and blood glucose lowering effect. It should be noted that changes in blood insulin were not significant. It is possible that PAZ may contain some molecule(s) able to act on basal metabolic rate (BMR), thus increasing energy expenditure and leading to consumption of excess fat stores. This hypothesis could be assessed in a further study using calorimetry. Appetite reducers in some cases could be linked to reduced energy intake; however, it is unlikely to be the case with the use of PAZ, since most participants reported an increased rather than reduced appetite. The reduction in inflammation induced by PAZ, as evidenced by strongly decreased levels of C-reactive protein (mean change -6.20 mg/L; -57%), should be considered here, as it may be responsible for improving blood sugar profiles through a reversal of the autoimmune component of insulin resistance. With regard to lipid profile, strong hypolipidemic effects of PAZ in the test group over a 10-week period suggest its potential protection against cardiovascular diseases through a reduction in total cholesterol (mean -64.8 mg/dL; -32%), LDL-cholesterol (mean -40.6 mg/dL; -30%) and triglycerides (mean -18.8 mg/dL; -11%), and an increase in HDL-cholesterol (mean 15.4 mg/dL; +42%). 1 out of 2 test subjects (n = 11) observed an increase of ≥15 mg/dL in HDL-cholesterol over 10 weeks, and nearly a third (n = 7) saw an increase of 20 mg/dL or more. All subjects taking ProAlgaZyme (vs. only 11% of subjects on placebo) saw an increase of ≥1 mg/dL in HDL-C. An increase in HDL-cholesterol alone has been shown to impart possible health benefits in overweight and obese people and reduce the risk for cardiovascular disease [20,21]. This increase in HDL-C coupled with a decrease in the concentrations of LDL-C and triglycerides could lead to a lowering of atherogenicity and therefore a significant reduction in the potential incidence of coronary heart disease [22]. Ten weeks of treatment with ProAlgaZyme brought about a mean reduction in total cholesterol of over 60 mg/dL, while only a 23.2 mg/dL (0.6 mmol/L) reduction of serum cholesterol has been shown to correlate with a 54% reduction of risk [23]. Additionally, half of test subjects observed a decrease in LDL-C of at least 36 mg/dL. PAZ may achieve these effects through interaction with enzymes involved in cholesterol biosynthesis, by catalysis of LDL-cholesterol conversion into bile salts, or by other mechanisms. Since a recruitment criterion in the study was hyperlipidemia, these results show clearly that PAZ could be used successfully for the relief of dyslipidemia. This improvement in lipid profile, coupled with a decrease in inflammatory markers, could also assure a protection against lipid plaque buildup and rupture in artery walls, and therefore against cardiovascular disease and hypertension, as suggested in the study by a decrease in blood pressures in PAZ patients. Yet, we cannot exclude the presence of hypotensive molecules in the infusion. Reductions in C-reactive protein, RBC sedimentation rate and Interleukin-6 (IL-6) were significantly greater in the PAZ group compared to placebo, suggesting that PAZ also has a preventive effect on the pro-inflammatory state generally linked to metabolic syndrome, and may be useful for the treatment of other inflammatory disorders. 100% of test subjects (n = 22) observed a 20% or more reduction in CRP (≥2.0 mg/L), and half of these (n = 11) experienced a reduction of 55% or more (≥6.0 mg/L). Collectively, these results support the use of PAZ for weight loss and metabolic and lipid imbalances, and warrant further investigation of the potential effects of ProAlgaZyme on cardiovascular health.

## Conclusion

ProAlgaZyme (PAZ) oral algae infusion demonstrated multiple beneficial effects on the various aspects of metabolic syndrome and may significantly promote cardiovascular health in these subjects. ProAlgaZyme was able to reduce body weight and BMI in overweight and obese persons, and trigger an improvement in lipid profile characterized by a decrease of blood total cholesterol, LDL-cholesterol and triglycerides, coupled with an increase in HDL-cholesterol. In addition, the infusion reduced fasting blood glucose and brought about a diminution of markers of inflammation. The infusion was well-tolerated and no side effects were noted in the study. The changes obtained after 10 weeks were significant (p < 0.05 or better). These new findings warrant further exploration into the active components in ProAlgaZyme and its potential newly discovered metabolic and cardiovascular health benefits.

## Methods

### Recruitment of participants for study

Recruitment of participants for the study was done from the Laboratory of Nutrition and Nutritional Biochemistry (LNNB) database, as well as by radio and poster adverts in the Yaoundé area and at the University of Yaoundé I Teaching Hospital. The recruitment adverts specifically targeted participants with relatively high total and LDL-cholesterol, as well as those with higher than normal fasting blood glucose levels. This was achieved through an initial free screening program organized by the LNNB. A physician examined participants to ascertain their inclusion into the study. Metabolic syndrome was diagnosed using the American Heart Association criteria for the disease, which is described as a combination of the following symptoms:

• Abdominal obesity (excessive fat tissue in and around the abdomen)

• Atherogenic dyslipidemia (blood fat disorders – high triglycerides, low HDL cholesterol and high LDL cholesterol – that foster plaque buildups in artery walls)

• Elevated blood pressure

• Insulin resistance or glucose intolerance (the body can't properly use insulin or blood sugar)

• Pro-thrombotic state (e.g., high fibrinogen or plasminogen activator inhibitor-1 in the blood)

• Pro-inflammatory state (e.g., elevatedC-reactive protein in the blood)

### Subject selection

#### Inclusion Criteria

Participants were male or female, ages 25–60, and gave their written informed consent. In addition, participants met at least 3 of the following criteria:

• BMI ≥ 30 kg/m^2^

• HDL Cholesterol of < 40

• Triglycerides > 150 mg/dl

• Fasting blood glucose > 100 mg/dl

• Blood pressure > 130/85 mm Hg

• Total Cholesterol of > 200 mg/dl

• LDL Cholesterol of > 160 mg/dl

• Interleukin 6 (IL-6) > 5 pg/mL

And were excluded if they:

• Were morbidly obese: BMI > 40 kg/m^2^

• Were taking any cholesterol-lowering medications 30 days prior to the start of enrollment and during the course of the study.

• Had been enrolled in another clinical study in the past 6 months.

• Were pregnant, actively infected, on medication that interfered with healing (for example, steroids), were inflicted with systemic disease such as AIDS, HIV, active hepatitis or active malignancy (clinical signs within the past 5 years), or suffered from diabetes mellitus requiring daily insulin management.

Suitable participants were divided into two groups (Table 2). A total of 60 overweight or obese participants, ages between 25 and 60 years, were selected from a group responding to radio and poster adverts. The study was conducted in a double-blind fashion, and patients were randomly assigned to a treatment. The two groups were studied in parallel for a total of 10 weeks.

**Table 2:** Participant groups.

	**Participant Characteristic**	**Treatment**	**Number of Participants**
Group 1	Metabolic syndrome	Placebo	30
Group 2	Metabolic syndrome	ProAlgaZyme	30

The BMI of participants ranged from 28.3 to 38.1, and their weights ranged from 74.3 to 107.6 kg. After physical examination, which included measurement of blood pressure, participants with unusually elevated fasting blood glucose levels, pregnant or lactating, as well as those on any form of weight, cholesterol, or inflammation-reducing medication were excluded from the study. Also excluded were participants involved in intense exercise programs, those who had medical conditions known to affect serum lipids or those who had a history of drug or alcohol abuse.

The study was approved by the University of Yaoundé Internal Review Board. The purpose, nature and potential risks of the study were explained to all participants, who gave their written informed consent before participation. The study was conducted in accordance with the Helsinki Declaration (1983 version).

### Study material and dosing regimen

ProAlgaZyme freshwater algae infusion (PAZ) and water placebo were obtained from Health Enhancement Products, Inc., Scottsdale, Arizona, USA. Materials had identical taste and appearance, and were packaged and labeled identically.

Baseline values were established in the first week, and ProAlgaZyme or placebo was administered orally for a total of 10 weeks. Each participant received 4 oz. of PAZ or placebo per day, divided into 4 equal doses of 1 oz. taken at breakfast, lunch, dinner and bedtime.

### Sample collection, parameters to be measured and timelines

At each of four time points, the testing protocol followed the order: blood sample collection, anthropometric measurements, administration of questionnaires. Time points were as follows: at the start (T = 0), as well as at 4, 8 and 10 weeks, i.e. T = 4, T = 8 and T = 10. The various anthropometric and biochemical parameters were analyzed using different methods and analytical kits as outlined below.

### Anthropometry

Body weight and percent body fat were determined in overnight-fasted participants using a Tanita™ scale. Height was measured with a stadiometer to the nearest 0.5 cm, and BMI was expressed as weight (kilograms) per height (meters) squared.

### Blood pressure

Blood pressure was measured on three different occasions using a mercury sphygmomanometer. After measuring blood pressure on day 1, a second measurement was obtained about a week later, and a third measurement 1 to 3 days later. On each occasion at least 2 readings were taken and the mean value recorded. An appropriate adult cuff was applied 2 to 3 cm above the antecubital fossa of the right arm. Blood pressure was measured to the nearest 2 mm Hg, reading the calibration below the meniscus with the subject in the sitting position. Systolic and diastolic blood pressures were read at the 1st and 5^th ^Korotkoff phases, respectively. The mean of the 3 blood pressure values obtained from the 3 visits was recorded as the subject's true blood pressure. Hypertension was defined as systolic and diastolic blood pressures equal to or more than 135 mm Hg and 85 mm Hg, respectively [24].

### Sample collection and treatment

Fasting blood (5 ml) was collected by venous puncture from subjects after an overnight fast at each of the time points, and distributed into heparinized tubes. After centrifugation at 3000 g for 10 minutes at 4°C plasma was stored in 0.5 ml aliquots at -80°C, and analyzed within 2 weeks.

### Biochemical analyses of plasma

Plasma total cholesterol was measured by the cholesterol oxidase method [25] while plasma triglyceride was determined as described by Bucolo and David [26]. HDL cholesterol was determined using a heparin manganese precipitation of Apo B-containing lipoproteins [27]. LDL cholesterol was calculated using the Friedewald formula [28]. Blood glucose was determined using the methods of Trinder [29]. CRP was determined using a turbidimetric method [30].

### Biochemical and physiological parameters

Parameters relating to metabolic syndrome were measured at four time points (T = 0, 4, 8 & 10 wk) (Table 3) in treatment and placebo group subjects.

**Table 3:** Biochemical and physiological parameters

Baseline T = 0	T = 4 weeks	T = 8 weeks	T = 10 weeks
BMI	BMI	BMI	BMI
Body fat	Body fat	Body fat	Body fat
Systolic blood pressure	Systolic blood pressure	Systolic blood pressure	
Diastolic blood pressure	Diastolic blood pressure	Diastolic blood pressure	
Pulse rate	Pulse rate	Pulse rate	Pulse rate
Insulin	Insulin	Insulin	Insulin
Total cholesterol	Total cholesterol	Total cholesterol	Total cholesterol
LDL-cholesterol	LDL-cholesterol	LDL-cholesterol	
HDL-cholesterol	HDL-cholesterol	HDL-cholesterol	HDL-cholesterol
Triglycerides	Triglycerides	Triglycerides	
Fasting blood glucose	Fasting blood glucose	Fasting blood glucose	Fasting blood glucose
RBC sedimentation	RBC sedimentation	RBC sedimentation	
CRP	CRP	CRP	CRP
IL-6	IL-6	IL-6	IL-6
TNF-a	TNF-a	TNF-a	TNF-a

### Statistical analyses

Statistical Package for the Social Sciences (SPSS) [31] software was used for all statistical analysis. The data were presented as means ± (1.96*SEM) for a 95% confidence level. The statistical difference between samples was assessed by a student's t-test for normal distribution or the Mann-Whitney test for non-normal distribution, after ANOVA testing of all the groups showed that significant difference existed. Paired Student's t-test was carried out on the start and end values of the groups.

## Competing interests

Authors TI, TT, DH and MT declare competing interests. These authors have received salary and/or stocks from Health Enhancement Products, Inc., the company who financed the clinical study above and this manuscript, including the article-processing charge. Authors TT, MT and DH are inventors on patents associated with ProAlgaZyme, the test material used in this study, and these patents have been financed by Health Enhancement Products, Inc.

## Authors' contributions

JO and DM conceived, designed and coordinated the work, as well as drafted the manuscript; EE carried out analytical work; DK carried out analytical and statistical analyses of data; TT participated in the design and editing of the manuscript. All authors have read and approved the manuscript.

## Supplementary Material

Additional file 1 Individual participant data from placebo and PAZ treatment groups for parameters relating to metabolic syndrome over 10 weeks. The data provided represent the effects in 52 subjects of 10-week oral treatment with placebo or ProAlgaZyme (PAZ) on parameters relating to metabolic syndrome at T = 0, 4, 8 and 10 weeks. Click here for file
